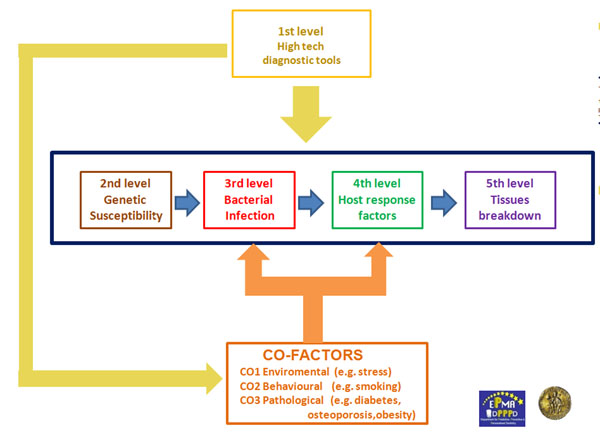# Predictive, preventive, personalised, participatory periodontology and implantology: the “5Ps” to approach the future

**DOI:** 10.1186/1878-5085-5-S1-A109

**Published:** 2014-02-11

**Authors:** Carlo Cafiero

**Affiliations:** 1 University of Naples “FEDERICO II”, Naples 80131, Italy

## 

In order to reconsider guidelines in dentistry, it is required to introduce new concepts of personalised patient diagnosis and treatment plan [1]. Worldwide dentists have to make a big cultural effort in changing the actual “reactive” therapeutic point of view, belonging to the last century, into a futuristic “predictive” one. The futuristic “5 Ps” (Predictive, Preventive, Personalised and Participatory Periodontology) focuses on the early integrated diagnosis (genetic, microbiology, biomarkers host- derived detection) and on the active role of the patient in which networked patients will shift from being mere passengers to responsible drivers of their health. We intend to propose five diagnostic levels (High tech diagnostic tools, Genetic susceptibility, Bacterial Infection, Host-response factors and tissues breakdown derived products) to be evaluated with the intention to obtain a clear picture of the vulnerability of the single individual for periodontitis in order to organise patients stratification in different categories of risk [2].

Five diagnostic levels are here proposed (High tech diagnostic tools, Genetic susceptibility, Bacterial Infection, Host-response factors and tissues breakdown derived products), in order to evaluate the vulnerability of the single individual for periodontitis to organise patients stratification in different categories of risk [3]. Lab-on-a-chip technology may soon become an important part of efforts to improve worldwide periodontal health in developed nations as well as in the underserved communities, resource-poor areas and poor countries. The use of LOCs devices for periodontal inspection will allow patients to be screened for periodontal diseases in settings other than the periodontist practice, such as at a general practitioners, general dentists or dental hygienists. Personalised therapy tailored respect to the particular medical reality of the specific stratified patient will be the ultimate target to be realised by “5Ps” approach. A long distance has to be covered to reach the above targets but the pathway has already been clearly outlined. Lab-on-a-chip technology may soon become an important part of efforts to improve worldwide periodontal health [4]. In developed nations, the most highly valued qualities for portable, easy-to-use diagnostic tools include speed, sensitivity, and specificity; while in the underserved communities, resource-poor areas and poor countries the goal of researchers is to create micro fluidic chips that will allow healthcare providers in poorly equipped hospitals. The use of LOCs devices for periodontal inspection will involve less education than current diagnostic procedures and allow patients to be screened for periodontal disease in settings other than the periodontist practice, such as at a general practitioners, general dentists or dental hygienists. All these benefits make the Lab-on-a-chip technology ideal for Predictive, Preventive, Personalised and Partecipatory Periodontology. “The 5Ps” represent the future of our profession. Personalised therapy tailored respect to the particular medical reality of the specific stratified patient will be the ultimate target to be realised by “5Ps” approach. A long distance has to be covered to reach the above targets but the pathway has already been clearly outlined: it is *“Time for new guidelines in advanced healthcare”* in dentistry too [5].

## Illustration materials

5Ps flow-chart. Five levels characterise a futuristic approach for periodontal diagnosis. The first level is represented by high tech diagnostic tools such as lab-on-chip (LOC) and cone beam computed tomography (CBCT). In the next future LOC will be able to give us genetic, microbiological and host derived information in real time. Co-factors (e.g. diabetes, osteoporosis etc.) will be detected by the use of dedicated high-tech chair side diagnostic tools. Moreover, a detailed bone tissue morphology is revealed by low radiation digital computed tomography which offers a digital volume composed of three dimensional voxels that can then be manipulated with specialised software. The second level will provide useful information about genetic susceptibility of the single patient, while the third level will give us the presence of causative bacterial factors in dental plaque. Finally, host derived biomarkers (host response factors and factors derived from periodontal tissue breakdown) will be chair-side detected in order to early intercept periodontal destruction.

**Figure 1:**